# Reply to Cabello et al., “Aquaculture and *mcr* Colistin Resistance Determinants”

**DOI:** 10.1128/mBio.01629-18

**Published:** 2018-08-28

**Authors:** Yingbo Shen, Wenjuan Yin, Dejun Liu, Jianzhong Shen, Yang Wang

**Affiliations:** a Beijing Advanced Innovation Center for Food Nutrition and Human Health, College of Veterinary Medicine, China Agricultural University, Beijing, China; Indiana University Bloomington

**Keywords:** aquaculture, colistin, *mcr-1*

## REPLY

We thank Cabello et al. (1) for taking the time to offer comments regarding our paper “Novel Plasmid-Mediated Colistin Resistance Gene *mcr-3* in Escherichia coli” (2). We agree that aquaculture is closely associated with the emergence of mobile colistin resistance (*mcr*) genes and have further insights on this hypothesis.

**FIG 1:**
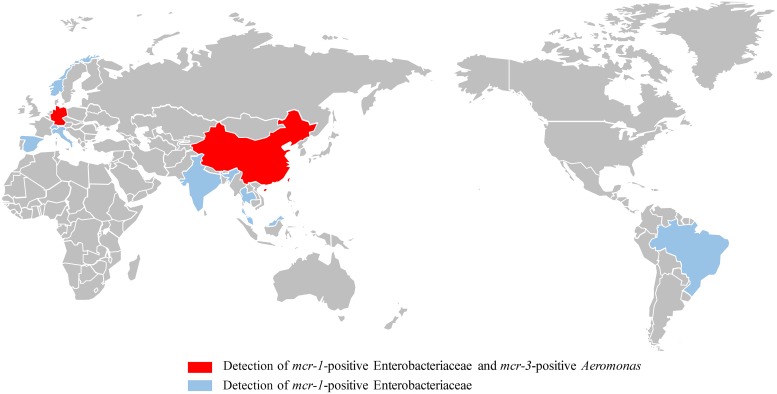
Global distribution of *mcr*-positive samples from aquatic environments.

To date, eight mobile colistin resistance genes (*mcr-1* to *mcr-8*), as well as several variants, have been characterized, mainly in *Enterobacteriaceae* from animals, humans, and the environment (3, 4). However, there is strong evidence supporting the hypothesis that these *mcr* determinants originated from aquatic environments. First, *mcr* genes are increasingly being identified either in *Enterobacteriaceae* from aquatic environments (*mcr-1* and *mcr-3*, Fig. 1) (5–7) or in aquatic bacterial species, such as *mcr-3* (8), *mcr-5* (9), and *mcr-7.1* (D. Liu, unpublished data) in *Aeromonas* species. Second, the flanking regions of *mcr* genes in Escherichia coli and Klebsiella pneumoniae strains either originate from aeromonads or show high amino acid identities to corresponding regions in *Aeromonas* species. These regions include several transposon (Tn) elements (Tn*As2* and Tn*As3*, adjacent to *mcr-3* in E. coli) (2), insertion sequences (IS*Aeca6* and IS*As13*, close to *mcr-3*) (10), and functional genes (diacylglycerol kinase gene *dgkA*, near *mcr-3* and *mcr-7.1*, and plasmid replicase-encoding gene *rep*, close to *mcr-5*) (9, 11). Third, other than *mcr-1* and *mcr-2*, all of the newly identified *mcr* genes encoding plasmid-mediated phosphoethanolamine transferases show significant amino acid sequence similarity to MCR proteins from aquatic bacteria, including MCR-3, MCR-3-like, and MCR-7.1 from *Aeromonas* species; MCR-4 from *Shewanella* species; and MCR-5 from *Legionella* species (3).

Our recent study on anthropogenic factors associated with a high incidence of *mcr-1* carriage in human-derived bacterial isolates across China also strongly supports the hypothesis that aquaculture may play a vital role in the dissemination of *mcr-1* (12). It revealed that geographic zones with limited aquaculture industries had significantly lower odds (odds ratio [OR] = 0.5; 95% confidence interval [CI], 0.3 to 0.7) of *mcr-1* carriage in human isolates than those with greater aquaculture activity. In addition, populations who ate smaller volumes of aquatic food (≤100 g/day) also had lower odds (OR = 0.6; 95% CI, 0.5 to 0.7) of human *mcr-1*-positive bacterial colonization. Although colistin is not approved for use in aquaculture in China, aquatic products and their related environments are highly likely to be contaminated with residual colistin from agricultural runoff, as oral administration of colistin was approved for the purpose of growth promotion in pig and poultry farming prior to April 2017. The high stability of colistin in water (13) exacerbates the persistence and dissemination of *mcr-1* and its host bacteria in aquatic environments by providing a selective pressure, leading to the possibility of further *mcr-1* contamination of aquatic foods (12).

At present, all policies worldwide aimed at restricting the use of colistin are mainly focused on farm animals (particularly pigs and poultry), while its use in aquaculture has rarely been acknowledged. Therefore, as also proposed by Cabello et al., urgent action is needed to assess and control colistin usage in aquaculture practice, especially in countries with well-developed freshwater aquaculture industries. We hope that our findings will help to encourage such investigations.
